# Supplementary material for: Acetone application for administration of bioactive substances has no negative effects on longevity, fitness, and sexual communication in a parasitic wasp
Source: PLoS One. 2021 Jan 20;16(1):e0245698. doi: 10.1371/journal.pone.0245698 (PMC7816986; doi:10.1371/journal.pone.0245698)
Supplement: Attachment — Submitted filename: Response to Reviewers.docx [file pone.0245698.s003.docx]

Answers to each point indicated by ## and red font.

Remark by the editorial office: “We note that you have included the phrase “data not shown” in your manuscript. Unfortunately, this does not meet our data sharing requirements. PLOS does not permit references to inaccessible data. We require that authors provide all relevant data within the paper, Supporting Information files, or in an acceptable, public repository. Please add a citation to support this phrase or upload the data that corresponds with these findings to a stable repository (such as Figshare or Dryad) and provide and URLs, DOIs, or accession numbers that may be used to access these data. Or, if the data are not a core part of the research being presented in your study, we ask that you remove the phrase that refers to these data”

##The phrase was not essential for the paper and therefore removed.##

Reviewer #1: This work describes experiments to characterize and assess the effects of acetone, a frequently used solvent vehicle in insect research, on parameters of insect reproduction in parasitic wasps of the genus Nasonia. The work is thorough and presented clearly. It’s refreshing to see solvent toxicity examined with so much attention to detail. Beyond asking how acetone might affect the wasps’ fitness, the researchers also investigate the efficacy of acetone in evaluating the effects of various biomolecules. This is quite informative, and the methods presented could be very useful for future studies involving Nasonia. I have a few comments mostly relating to style and clarity, but also, more details justifying the choice of stats should be added. See below.

IntroL35-37: Two sentences on the importance of insects to justify studying them feels rushed. Perhaps a few more lines dedicated to describing why insect research in important are merited.

##A few sentences explaining the importance of insects in more detail have been added with some additional references. ##

L121-124: More background on the biological questions being addressed by the application experiments in the intro would be beneficial.

##Some additional sentences introducing the application examples have been added to the introduction.##

MethodsL193: Is this the only component or just the most important sex pheromone? Why was this the only component examined?

##In contrast to *N. vitripennis*, *N. giraulti* has only one stereoisomer of 5-hydroxy-4-decanolide in its pheromone (RS-HDL). Information is now given in the introduction. The minor component 4-methylquinazoline was detectable in the samples but not quantifiable by GC/MS.##

L208: 20-OH-E and JH and the research that led to this application test should be discussed in the intro.

##done##

L225: The same here for pheromone biosynthesis.

##done##

L243: remove “do”

##not done##

L243: Again, it’s important to mention ahead of the methods why the author chose to perform these experiments.

##introduction was modified##

L280: I am curious why nonparametric approaches were selected. I also wonder what a more inclusive model for certain analysis might yield. For example, if you examine interactive effects of age and treatment, would you see any interactions? A little explanation for the choices made would be helpful.

##Not all data sets met the assumptions of parametric statistics (normal distribution). That is why we chose non-parametric methods. Information is now given.##
